# A New Vision Measurement Technique with Large Field of View and High Resolution

**DOI:** 10.3390/s23146615

**Published:** 2023-07-23

**Authors:** Yong Li, Chenguang Liu, Xiaoyu You, Jian Liu

**Affiliations:** 1 Research Center of Advanced Microscopy and Instrumentation, Harbin Institute of Technology, Harbin 150001, China; ly_1987621@163.com (Y.L.); youxiaoyuhit@hit.edu.cn (X.Y.); 2 Research Center of Basic Space Science, Harbin Institute of Technology, Harbin 150001, China; chenguang_611@126.com

**Keywords:** visual technology, confocal technology, large field of view, three-dimensional resolution, confocal scanning, optical measurement

## Abstract

The three-dimensional (3D) displacement resolution of conventional visual measurement systems can only reach tens of microns in cases involving long measuring distances (2.5 m) and large fields of view (1.5 m × 1.5 m). Therefore, a stereo vision measurement technology based on confocal scanning is proposed herein. This technology combines macroscopic visual measurement technology with confocal microscopic measurement technology to achieve a long measuring distance, a large field of view, and micron-level measuring resolution. First, we analyzed the factors affecting the 3D resolution of the visual system and developed a 3D resolution model of the visual system. Subsequently, we fabricated a prototype based on the resolution model and the proposed stereo vision measurement technology. The 3D displacement resolution measurement results in the full field of view show that the displacement resolutions of the developed equipment in the x-, y-, and z-directions can reach 2.5, 2.5, and 6 μm, respectively.

## 1. Introduction

Stereoscopic vision technology [1,2,3,4,5,6] is a technology that mimics human eye imaging. It uses two or more cameras to image the same object from different orientations and then calculates the parallax through stereo matching. Finally, it calculates the three-dimensional (3D) geometric information of the object based on the calibrated internal and external parameters of the visual system. Visual technology is widely used and investigated owing to its characteristics of zero contact, high speed, and high measurement accuracy. In recent decades, visual technology has been increasingly used in many fields, such as robot vision, aerial surveying, medical imaging, and industrial testing.

However, most researchers of visual technology tend to focus on improving the measurement accuracy and success rate by investigating (i) different calibration algorithms [7,8,9,10,11] to improve the calibration accuracy, (ii) different matching algorithms to improve the matching accuracy [12,13,14,15], and (iii) three-dimensional reconstruction algorithms to improve the reconstruction accuracy [16,17,18]. In addition, researchers have focused on expanding the application scenarios [19,20]. Some researchers have studied the relationship between visual system structural parameters and measurement accuracy [21,22,23,24,25]. Resolution, a crucial indicator in the field of measurement, is typically disregarded in visual technology. The resolution of vision technology is defined as the minimum displacement of an object that can be recognized using vision technology. This is primarily because resolution is not an advantage of visual measurement technology, nor is it prioritized in most application fields of visual technology. Therefore, resolution is typically not considered in visual technology. However, in some application scenarios, not only the main indicators of visual technology—such as field of view (FOV) and accuracy—but also resolution must be considered. Gong et al. [26] and Li et al. [27] analyzed the effects of individual structural parameters of a visual system on its resolution; however, they did not disclose a method to overcome the inherent resolution limitations of the visual system. Under normal circumstances, when the test distance exceeds 2.5 m and the FOV exceeds 1.5 m × 1.5 m, the resolution of the conventional vision system is typically tens or hundreds of microns.

Confocal scanning imaging technology [28,29,30] is a two-dimensional (2D) optical measurement technology that can realize 3D measurements of objects by combining axial scanning with axial positioning technology. The FOV of confocal technology is typically in the order of millimeters or microns. Confocal technology uses the three-point conjugation principle (point illumination, point detection, and point object) to scan and image objects point by point. Unlike the case of conventional array detection technology, the sampling interval in confocal technology depends on the scanning and sampling frequencies. Based on the existing scanning and sampling equipment, the sampling interval can reach nanometers or sub-nanometers. Confocal technology is a micro measurement technology that offers FOV measurements not exceeding a few millimeters.

Herein, we propose a new vision measurement technology based on confocal scanning imaging that allows a large FOV and a high resolution to be achieved simultaneously in vision measurements. First, we develop a 3D resolution model of the visual system, through which the factors affecting the resolution of the visual system can be obtained. Subsequently, we combine confocal scanning technology with vision technology to propose a vision measurement technology based on confocal scanning imaging for the first time. This technology uses the point-scanning imaging characteristics of confocal technology to reduce the sampling interval and overcome the resolution limit of conventional vision technology.

## 2. Methods

### 2.1. Resolution Model of a Stereo Vision System

**Figure 1:**
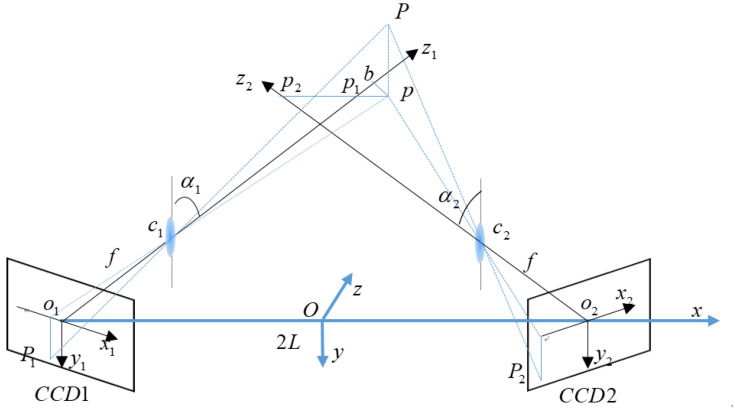
Schematic diagram of binocular vision.

A binocular stereo vision system typically comprises two identical monocular vision systems, the principle of which is illustrated in Figure 1. The binocular system comprises two monocular systems, and each monocular vision system comprises an optical imaging system (*c*_1_ or *c*_2_) and an imaging detector (CCD1 or CCD2). The optical imaging system (*c*_1_ or *c*_2_) is not necessarily a camera and can be another complex optical imaging system equivalent to a thin lens without considering aberration. Any type of imaging detector can be used, provided that it can capture the image of the object. The two optical systems are assumed to be identical. The image distance is f; the center distance (baseline distance) of the two imaging planes is 2*L*; and *o*_1_ and *o*_2_ are the intersection points between the optical axes of the left and right monocular imaging systems and the corresponding imaging plane, respectively. The world coordinate system, *O*-*xyz*, was constructed by considering the midpoint of *o*_1_*o*_2_ as the origin and the extension line as the *x*-axis. The optical axis inclinations of left and right monocular imaging systems are *α*_1_ and *α*_2_, respectively, where *α*_1_ = *α*_2_ = *α*. The imaging coordinate systems of the left and right monocular imaging systems are *o*_1_*x*_1_*y*_1_*z*_1_ and *o*_2_*x*_2_*y*_2_*z*_2_, respectively, with *o*_1_ and *o*_2_ as the origins, respectively. The optical axes of the left and right monocular imaging systems are *o*_1_*z*_1_ and *o*_2_*z*_2_, respectively. Point *P* is the point to be measured and is located in the FOV of the binocular stereo vision system. The imaging points on the two imaging planes are points P_1_ and P_2_, whose coordinates are (*x*_1_, *y*_1_, 0) and (*x*_2_, *y*_2_, 0), respectively. Point *p* is the intersection of point P projected vertically onto the *c*_1_*c*_2_*o*_1_*o*_2_ plane; hence, point *P* has the same *x*- and *z*-coordinates as point *p*. Point *p* forms a straight line parallel to *o*_1_*o*_2_ on the *c*_1_*c*_2_*o*_1_*o*_2_ plane, which intersects *o*_1_*z*_1_ and *o*_2_*z*_2_ at points *p*_1_ and *p*_2_, respectively. A perpendicular line that passes point *p* intersects *o*_1_*z*_1_ at point *b*.

Before analyzing the factors affecting the resolution of the visual system, the definition of resolution should be presented. We define the resolution of a binocular stereo vision system as the minimum displacement of an object that can be effectively recognized by the visual system. Based on the characteristics of the visual system, resolution can be defined as the displacement of an object when a perceptible change occurs in the imaging plane of the left or right monocular system.

As shown in Figure 1, the coordinate of object point *P* (*x*, *y*, *z*) in the imaging coordinate system *o*_1_*x*_1_*y*_1_*z*_1_ of the left visual system is (*s*_1_ × cosα_1_, *y*, *z* × sec*α*_1_ + *s*_1_ × sin*α*_1_), and the coordinate of object point *P* in the imaging coordinate system *o*_2_*x*_2_*y*_2_*z*_2_ of the right visual system is (*s*_2_ × cos*α*_2_, *y*, *z* × sec*α*_2_ − *s*_2_ × sin*α*_2_), where:

(1) $$\begin{array}{l} s_{1}=p_{1}p=x+L-z\tan\alpha_{1}\\ s_{2}=p_{2}p=x-L+z\tan\alpha_{2} \end{array}$$

Based on the similar triangle principle, we obtain:

(2) $$\begin{array}{l} \frac{-x_{1}}{s_{1}\cos\alpha }=\frac{f}{z\sec\alpha +s_{1}\sin\alpha -f}=\frac{y_{1}}{-y}\\ \frac{-x_{2}}{s_{2}\cos\alpha }=\frac{f}{z\sec\alpha -s_{2}\sin\alpha -f}=\frac{y_{2}}{-y} \end{array}$$

Based on the definition of the resolution of the visual system, when point *P* propagates along any of the three directions (*x*, *y*, or *z*), the coordinates of the image points in the left and right cameras change accordingly. If the coordinate changes of one of the image points can be recognized, then it indicates that the vision system can distinguish changes in the position of point *P*, which is the resolution. The 3D resolutions of the visual system are independent of each other; therefore, we can calculate the resolution in three directions. The resolutions of the visual system in the *x*-, *y*-, and *z*-directions are denoted as Δ*X*, Δ*Y*, and Δ*Z*, respectively.

Suppose that point *P* propagates only slightly along the *x*-direction. In this case, to solve the displacement of image points in the left and right visual systems, the coordinates of point *P* should first be represented by the left and right imaging coordinates. By substituting Equation (1) into Equation (2), we obtain:

(3) $$x=\frac{-(L\sin\alpha +z\cos\alpha -f)x_{1}-Lf\cos\alpha +zf\sin\alpha }{x_{1}\sin\alpha +f\cos\alpha }$$

(4) $$x=\frac{y_{1}\left(f\cos\alpha -z\right)-fy\cos\alpha }{y_{1}\sin\alpha \cos\alpha }-L+z\tan\alpha$$

(5) $$x=\frac{-(L\sin\alpha +z\cos\alpha -f)x_{2}+Lf\cos\alpha -zf\sin\alpha }{-x_{2}\sin\alpha +f\cos\alpha }$$

(6) $$x=\frac{y_{2}\left(-f\cos\alpha +z\right)+fy\cos\alpha }{y_{2}\sin\alpha \cos\alpha }+L-z\tan\alpha$$

When *P* propagates only along the *x*-direction and does not change along the *y*- and *z*-directions, the derivatives of Equations (3)–(6) with respect to *x*_1_, *y*_1_, *x*_2_, and *y*_2_ can be obtained as follows:

(7) $$\frac{dx}{dx_{1}}=\frac{(z-f\cos\alpha )f}{{\left(x_{1}\sin\alpha +f\cos\alpha \right)}^{2}}=\frac{{\left[z\cos\alpha +(x+L)\sin\alpha -f\right]}^{2}}{(z-f\cos\alpha )f}$$

(8) $$\frac{dx}{dx_{2}}=\frac{-(z-f\cos\alpha )f}{{\left(-x_{2}\sin\alpha +f\cos\alpha \right)}^{2}}=-\frac{{\left[z\cos\alpha -(x-L)\sin\alpha -f\right]}^{2}}{(z-f\cos\alpha )f}$$

(9) $$\frac{dx}{dy_{1}}=\frac{fy}{y_{1}{}^{2}\sin\alpha }={\frac{\left[-z\cos\alpha -\left(x+L\right)\sin\alpha +f\right]}{fy\sin\alpha }}^{2}$$

(10) $$\frac{dx}{dy_{2}}=\frac{-fy}{y_{2}{}^{2}\sin\alpha }=-{\frac{\left[-z\cos\alpha +\left(x-L\right)\sin\alpha +f\right]}{fy\sin\alpha }}^{2}$$

Assuming that the left and right visual systems are identical, the minimum perceptible change on the image plane of the left and right visual systems is Δ*w*. Based on the definition of resolution, the resolution in the *x*-direction can be written as:

(11) $$\Delta X=\min\left(\left|\frac{dx}{dx_{1}}\right|\cdot \Delta w,\left|\frac{dx}{dx_{2}}\right|\cdot \Delta w,\left|\frac{dx}{dy_{1}}\right|\cdot \Delta w,\left|\frac{dx}{dy_{2}}\right|\cdot \Delta w\right)$$

Here, Δ*w* is a pixel without considering the subpixel algorithm. However, with the development of the subpixel algorithm, Δ*w* can be one-tenth of a pixel or even smaller, where *w* denotes the sampling interval.

Similarly, for the resolutions in the *y*- and *z*-directions, the imaging coordinate system of the left and right visual systems is used to represent the *y*- and *z*-coordinates of point *P*, respectively, and the derivative can be obtained as follows:

(12) $$\frac{dy}{dy_{1}}=\frac{-(x+L)\sin\alpha -z\cos\alpha +f}{f}$$

(13) $$\frac{dy}{dy_{2}}=\frac{(x-L)\sin\alpha -z\cos\alpha +f}{f}$$

(14) $$\frac{dz}{dx_{1}}=\frac{(x+L-f\sin\alpha )f}{{\left(-x_{1}\cos\alpha +f\sin\alpha \right)}^{2}}=\frac{{\left((x+L)\sin\alpha +z\cos\alpha -f\right)}^{2}}{(x+L-f\sin\alpha )f}$$

(15) $$\frac{dz}{dx_{2}}=\frac{(x-L+f\sin\alpha )f}{{\left(x_{2}\cos\alpha +f\sin\alpha \right)}^{2}}=\frac{{\left(-x\sin\alpha +L\sin\alpha +z\cos\alpha -f\right)}^{2}}{\left(-x+L-f\sin\alpha \right)f}$$

(16) $$\frac{dz}{dy_{1}}=\frac{fy}{y_{1}{}^{2}\cos\alpha }=\frac{{\left[-(x+L)\sin\alpha -z\cos\alpha +f\right]}^{2}}{fy\cos\alpha }$$

(17) $$\frac{dz}{dy_{2}}=\frac{fy}{y_{2}{}^{2}\cos\alpha }=\frac{{\left[(x-L)\sin\alpha -z\cos\alpha +f\right]}^{2}}{fy\cos\alpha }$$

Subsequently, the resolutions in the *y*- and *z*-directions of the visual system are expressed as:

(18) $$\Delta Y=\min\left(\left|\frac{dy}{dy_{1}}\right|\cdot \Delta w,\left|\frac{dy}{dy_{2}}\right|\cdot \Delta w\right)$$

(19) $$\Delta Z=\min\left(\left|\frac{dz}{dx_{1}}\right|\cdot \Delta w,\left|\frac{dz}{dx_{2}}\right|\cdot \Delta w,\left|\frac{dz}{dy_{1}}\right|\cdot \Delta w,\left|\frac{dz}{dy_{2}}\right|\cdot \Delta w\right)$$

A 3D resolution model of the visual system was developed. The theoretical resolution of any visual system can be obtained by combining the requirements of the FOV. Based on the model, the resolution of the visual system is not only related to the internal parameters of the visual system but also directly related to the external parameters of the visual system. The left and right visual systems are typically placed in parallel in an application environment featuring a large FOV. Therefore, the resolutions of a visual system with parallel optical axes are expressed as follows:

(20) $$\begin{array}{l} \Delta X=\min\left(\left|\frac{z-f}{f}\right|\cdot \Delta w,\left|\frac{f-z}{f}\right|\cdot \Delta w\right)\\ \Delta Y=\min\left(\left|\frac{z-f}{f}\right|\cdot \Delta w,\left|\frac{f-z}{f}\right|\cdot \Delta w\right)\\ \Delta Z=\min\left(\left|\frac{{\left(z-f\right)}^{2}}{\left(x+L\right)f}\right|\cdot \Delta w,\left|\frac{{\left(z-f\right)}^{2}}{\left(L-x\right)f}\right|\cdot \Delta w,\left|\frac{{\left(f-z\right)}^{2}}{fy}\right|\cdot \Delta w,\left|\frac{{\left(f-z\right)}^{2}}{fy}\right|\cdot \Delta w\right) \end{array}$$

Based on Equation (20), one can intuitively form the following conclusions: 1. The resolution of the parallel optical axis vision system is proportional to the sampling interval. 2. The resolution of the parallel optical axis vision system in the *x*- and *y*-directions remains the same over the entire FOV. 3. The *x*- and *y*-resolutions of the parallel optical-axis vision system are proportional to the test distance, whereas the *z*-resolution is proportional to the square of the test distance. 4. The *z*-direction resolution of the parallel optical-axis vision system is inversely proportional to the baseline distance.

**Figure 2:**
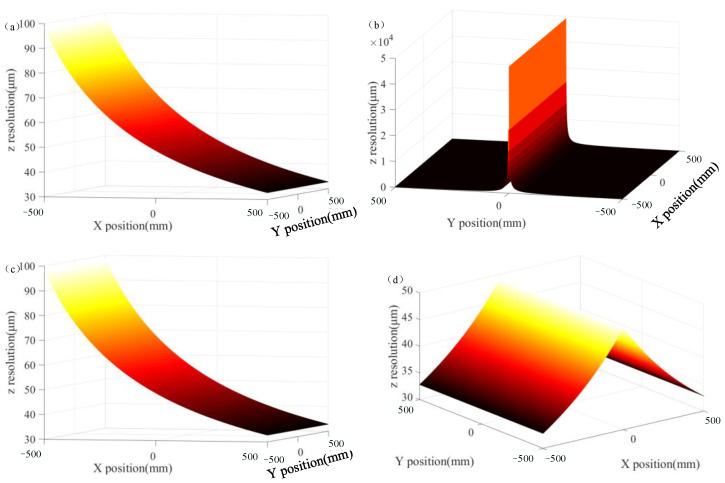
*Z*-resolution of parallel optical axis vision system. (**a**) The *x*-direction component of the *z*-direction displacement, (**b**) the *y*-direction component of the *z*-direction displacement, (**c**) *z*-direction resolution of the left system, and (**d**) *z*-resolution of parallel optical axis vision system.

The relationship between the resolution and FOV of the visual system can be shown more intuitively. The focal length of the left and right lens was assumed to be 16 mm; the sampling interval was 0.2 μm, i.e., Δ*w* = 0.2 μm; the FOV of the visual system was 1 m; the baseline distance, 2*L*, was 2 m; and the test distance of *z* was 2 m. Hence, the resolution of the monocular visual system in the *x*-direction was 24.8 μm. The resolution in the *y*-direction was also 24.8 μm, and the *z*-direction resolutions of the monocular and binocular vision systems are shown in Figure 2. As shown in Figure 2d, the binocular vision system with parallel optical axis indicated the worst resolution (49.2 μm) at the center of the FOV and the best resolution (32.8 μm) at the edge of the FOV. However, as shown in Figure 2c, the *z*-direction resolution of the monocular system was monotonic. Based on the left visual system as an example, the greater the distance from the optical axis of the left visual system, the higher the *z*-direction resolution. Figure 2a represents the change amount along the *x*_1_-direction on the image plane of the left visual system when point *P* changed along the *z*-direction, which is consistent with Figure 2c. Figure 2b shows the change in the value of point *P* along the *y*_1_-direction on the image plane of the left visual system when point *P* changed along the *z*-direction. When point *P* was in the center of the FOV, the displacement of point *P* in the *z*-direction did not reflect the change in the *y*_1_-direction. At the edge of the FOV, the minimum displacement of point *P* along the *z*-direction was 98.4 μm. In other words, when point *P* propagated along the *z*-direction, its image point was displaced along the *x*_1_- and *y*_1_-directions on the image plane, and the displacement along the *x*_1_-direction exceeded that along the *y*_1_-direction.

**Figure 3:**
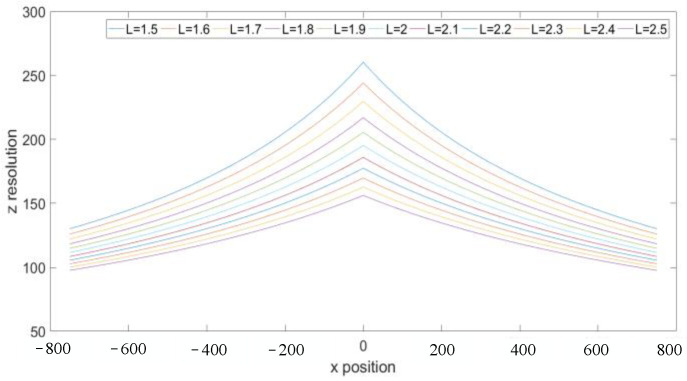
Effect of baseline distance on *Z*-direction resolution.

**Figure 4:**
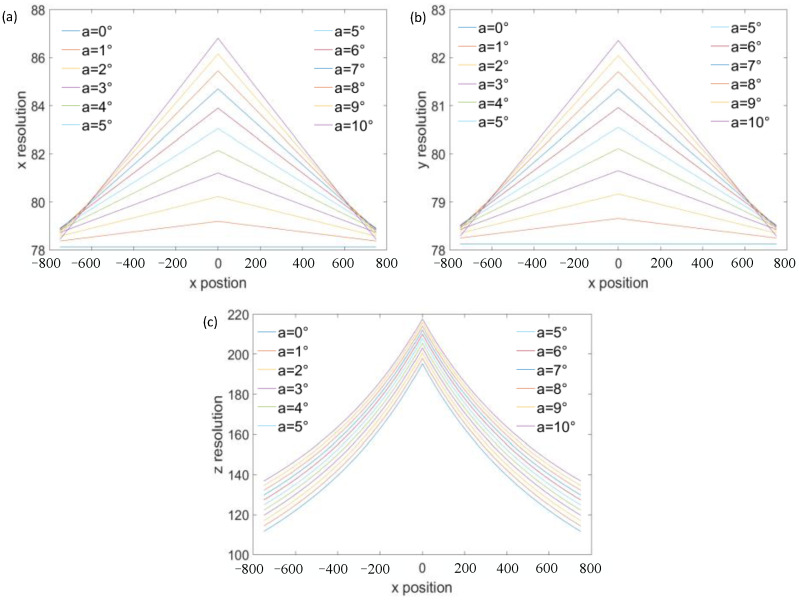
Effect of optical axis inclinations on resolution. (**a**) The effect of optical axis inclinations on *x*-direction resolution, (**b**) the effect of optical axis inclinations on *y*-direction resolution, and (**c**) the effect of optical axis inclinations on *z*-direction resolution.

To analyze the influence of other parameters on the resolution, we first give the parameters of the visual system as follows: the measurement distance is 2.5 m, the FOV is 1.5 m, the focal length is 16 mm, the sampling interval is 0.5 μm, and the size of the detector is infinite. The variable parameters are the optical axis inclination and the baseline distance of the visual system. First, assume that the optical axis inclination is 0° and the baseline distance changes from 1.5 m to 2.5 m. At this time, according to the resolution model, the resolution in the *x*- and *y*-directions is 78.125 μm, and the resolution in the *z*-direction is shown in Figure 3. It can be seen that the larger the baseline distance, the higher the *Z*-direction resolution. The baseline distance increased from 1.5 m to 2.5 m and the *z*-direction resolution increased from 260.4 μm to 156.3 μm. In theory, the resolution can be improved by increasing the baseline distance, but in practical applications, the increase in the baseline distance will inevitably lead to reduction in the FOV, so the resolution cannot be greatly improved by this method. We assume that the baseline distance is 2 m, and the optical axis inclination increases from 0° to 10°. At this time, the resolution of the visual system in *x*-, *y*-, and *z*-directions can be calculated according to the resolution model, as shown in Figure 4. As can be seen from the figure, the resolution decreases with the increase in the optical axis inclination. With the optical axis inclination increased from 0° to 10°, the resolution in the *x*-direction decreased from 78.125 μm to 86.81 μm, the resolution in the y-direction decreased from 78.125 μm to 82.36 μm, and the resolution in the *z*-direction decreased from 195.3 μm to 217.7 μm. Therefore, from the above analysis, it can be seen that for a visual system, the simplest and most effective way to improve the resolution is to reduce the sampling interval. There are two methods to reduce the sampling interval: one is to reduce the pixel size of the detector, and the other is to develop a new subpixel algorithm to subdivide the pixel size. But, at present, both methods are difficult to further break through.

**Figure 5:**
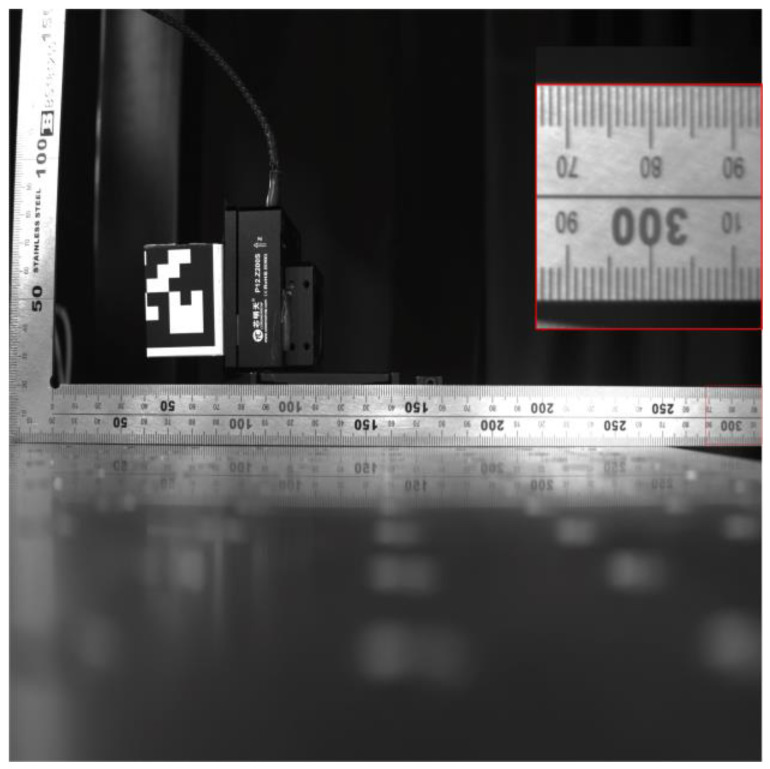
Calibration figure of the field of view.

**Figure 6:**
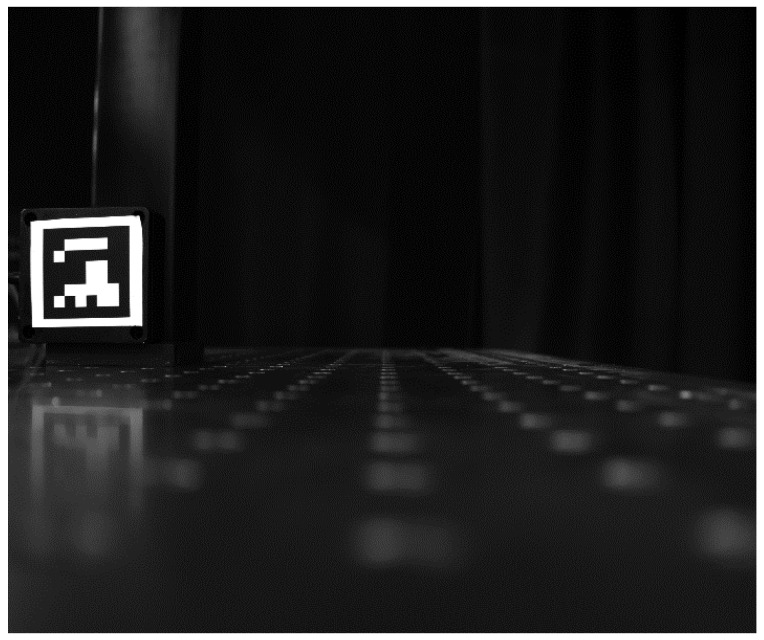
Location figure of the target.

In order to demonstrate the predictive capabilities of the 3D resolution model of a vision system, we constructed a vision system. The focal length of the lens used in the vision system is 8 mm (product model: HN-0816-5M-C2/3X). The imaging area of the imaging detector is 2/3 inch and the pixel size is 3.45 μm (product model: MER2-503-36U3M). The parameters of the displacement platform are as follows: the maximum stroke is 200 μm and the resolution is 20 nm. We first calibrated the field of view of the visual system, as shown in Figure 5. We can see that the FOV in the *x*-direction is about 315 mm, and we assume that the FOV in the *y*-direction is also 315 mm. The measured distance can be calculated as 315 × 8/8.8 ≈ 286 mm. Due to the limitation of the stroke of the displacement platform, we placed the target at the edge of the FOV of the vision system, as shown in Figure 6. At this time, the distance of the object from the optical axis was 96 mm. Assume that the minimum displacement recognized by the camera is 0.5 pixels, that is, 1.725 μm. The theoretical resolution calculated by the resolution model is 61.7 μm, 61.7 μm, and 183.7 μm in the *x*-, *y*-, and *z*-directions, respectively. Due to the limitation of the stroke of the displacement platform, in this experiment, the movement of our displacement platform is carried out in a reciprocating way—that is, it first moves *A* μm along the *x*-direction, and then moves −*a* μm along the *x*-direction, repeated 5 times. The displacement platform moves in the same way in the *y*- and *z*-directions. In the actual test, the distance we shift in the *x*- and *y*-directions is 62 μm, i.e., *a* = 62 μm. The distance shifted in the *z*-direction is 184.1 μm, that is, *a* = 184.1 μm. The displacement results calculated by image-matching algorithm based on ZNCC are shown in Table 1. As can be seen from the table, these displacements can be effectively identified, that is, the three-dimensional displacement resolution is (62 μm, 62 μm, 184.1 μm). The actual measured resolution is very close to the theoretical value, and only slightly larger than the theoretical value. This is mainly due to parameter error, noise, and other factors. Therefore, our model can be effectively applied to the traditional vision system to predict its resolution.

### 2.2. Principle of Visual Measurement Technology Based on Confocal Scanning Imaging

Based on the analysis presented in the previous section, the resolution of the visual system depends on the sampling interval; that is, the smaller the sampling interval, the higher the resolution of the visual system. However, in conventional vision systems, fixed detectors such as CCDs are used as imaging detection devices, and the pixel size is the sampling interval. Owing to factors such as processing technology and detector material, further reducing the pixel size of the detectors is challenging; consequently, the resolution of the conventional vision system cannot be easily improved. As shown in the section above, under the meter-level FOV, the resolution of the binocular system typically exceeds 10 µm. However, the structure of the vision technology is simple, the 3D displacement of the object can be calculated only by image matching, and the measurement FOV is relatively large.

Confocal imaging systems are typically used for microscopic measurements. These systems scan objects point by point and then perform single-point detection imaging for each scanning point. Therefore, the sampling interval for this technology depends on the scanning interval. The scanning interval can be adjusted based on the scanning frequency of the galvanometer and the sampling frequency of the data acquisition card. For existing data acquisition cards, the sampling rate can typically reach tens of megahertz, and a sampling interval in the micron or submicron level can be achieved under the meter-level FOV by matching the scanning frequency. Furthermore, confocal technology can achieve nanometer or sub-nanometer resolutions; however, it has a maximum FOV on the order of millimeters. A more detailed, confocal technique is a point-by-point scanning imaging technique. It illuminates the object point through a point light source and then collects the reflected light of the object point to image the object point. The original confocal technique is stationary like a fixed sensor, while the object moves in the plane through an x/y displacement platform to achieve scanning of the object. At this time, the three-dimensional information of the object cannot be obtained, and the axial displacement of the object cannot be obtained. In order to obtain three-dimensional information about the object, it is necessary to use the axial displacement platform to drive the object along the axis direction, and then use the x/y displacement platform to move the object in the plane and image the object, then repeat the above steps. In general, the number of axial movements is dozens or even hundreds of times. And then the three-dimensional information of the object is calculated through a certain algorithm. Moreover, confocal technology uses a point light source to illuminate an object and only illuminates one object point at a time. As the object moves in the *xy* plane, the object point illuminated by the point light source also changes. The confocal system collects the reflected signals of these different object points through the data acquisition card, and the sampling rate of the data acquisition card is very high, resulting in a very small interval between the two object points. Therefore, when the object point moves in the *xy* plane, confocal technology can be easily identified. When the object has axial displacement, confocal technology needs to obtain the three-dimensional information of the object before displacement, and then obtain the three-dimensional information of the object after displacement and calculate the axial displacement of the object through the relative change in the three-dimensional information. Therefore, this method is very slow, and with the development of technology, it is now common to use galvanometers to achieve two-dimensional scanning, and the two scanning methods are exactly the same. But axial scanning still requires the use of a displacement platform.

**Figure 7:**
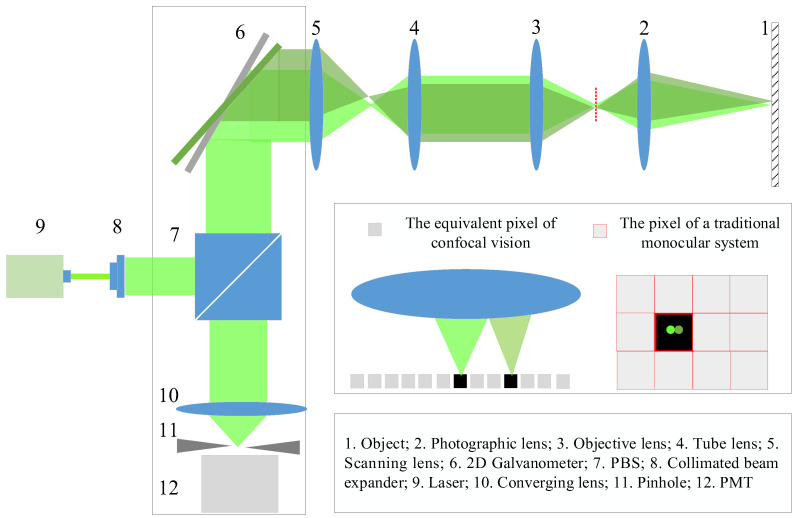
Schematic diagram of monocular vision system based on confocal scanning imaging.

Although visual and confocal measurement technologies are unrelated, we combined them to propose a technology based on confocal scanning. Our technology combines the advantages of the two technologies, using confocal technology to scan the object to improve the resolution, and then using vision technology to calculate the three-dimensional displacement of the object without axial scanning. A schematic illustration of the monocular system is shown in Figure 7. As shown in the schematic diagram, the technology is primarily composed of two components, i.e., a photographic lens, which serves to increase the measurement range of the system, and a confocal scanning imaging subsystem, which is composed of elements 3–12, as shown in the figure. A confocal scanning imaging module was used to replace the CCD imaging module of the conventional vision system, and a point-by-point scanning imaging method was used to realize the imaging measurement of the object. As shown in the schematic diagram, the imaging pixel was mapped to the object side, or the object was mapped to the image side. When the object point shifted slightly, the amount of movement was less than the corresponding size of the CCD pixel. Therefore, in the conventional visual system, the object point is imaged in the same pixel before and after displacement. Thus, even if the object point is displaced, the conventional visual system cannot recognize it. For vision technology based on confocal scanning imaging, the sampling interval can be reduced based on the sampling rate and scanning frequency, and submicron or smaller sampling intervals can be realized under a meter-level FOV. Therefore, for the confocal vision system, because the sampling interval was reduced, the object point was imaged at the 7th sampling interval (the first black pixel from left to right) before it shifted, and at the 10th sampling interval after it shifted. This implies that a slight displacement can be recognized.

We can treat the entire confocal module as an imaging detector whose role is to scan the image plane of the photographic lens. This is feasible because the diffraction effect is not taken into account when the resolution model is established, that is, the aperture of the photographic lens is treated as infinite. Therefore, imaging of the object by the photographic lens does not lose any information about the object, but only changes the size of the object. The scanning of the confocal module on the image plane is equivalent to a single-pixel detector scanning on the image plane. It is assumed that the focal length of the objective lens is *f*_2_, the tube lens is *f*_3_, the scanning lens is *f*_4_, the scanning angle range of the galvanometer is *θ*, the scanning frequency of the galvanometer is *m* Hz, and the sampling frequency of the data acquisition card is *n* Hz. According to the confocal scanning principle, the sampling interval is *m*/*n* × *θ*. Then, the minimum sampling interval on the front focal plane of the scanning lens is *f*_4_ × *m*/*n* × *θ*, and the sampling interval on the front image plane of the objective lens is:

(21) $$\Delta c=f_{4}\frac{f_{2}}{f_{3}}\frac{m}{n}\theta =\frac{f_{4}}{k}\frac{m}{n}\theta$$

where *k* represents the magnification of the objective lens and the tube lens, which is called the first-order magnification. When *k* = 1, the objective and tube lenses can be removed. Therefore, the 3D resolution model of vision measurement technology based on confocal scanning is expressed as follows:

(22) $$\begin{array}{l} \Delta X=\min\left(\left|\frac{d}{f}\right|\cdot \frac{f_{4}}{k}\frac{m}{n}\theta ,\left|\frac{d}{f}\right|\cdot \frac{f_{4}}{k}\frac{m}{n}\theta \right)\\ \Delta Y=\min\left(\left|\frac{d}{f}\right|\cdot \frac{f_{4}}{k}\frac{m}{n}\theta ,\left|\frac{d}{f}\right|\cdot \frac{f_{4}}{k}\frac{m}{n}\theta \right)\\ \Delta Z=\min\left(\begin{array}{l} \left|\frac{d^{2}}{\left(x+L\right)f}\right|\cdot \frac{f_{4}}{k}\frac{m}{n}\theta ,\left|\frac{d^{2}}{\left(L-x\right)f}\right|\cdot \frac{f_{4}}{k}\frac{m}{n}\theta \\ ,\left|\frac{d^{2}}{fy}\right|\cdot \frac{f_{4}}{k}\frac{m}{n}\theta ,\left|\frac{d^{2}}{fy}\right|\cdot \frac{f_{4}}{k}\frac{m}{n}\theta \end{array}\right) \end{array}$$

where *d* represents the test distance. The following can be seen from the formula: 1. The resolution of the vision measurement technology based on confocal scanning is proportional to the scanning frequency; that is, the smaller the scanning frequency, the higher the resolution of the system; 2. The resolution is inversely proportional to the sampling frequency; that is, the higher the sampling frequency, the higher the resolution of the system; 3. The resolution is proportional to the focal length of the scanning lens; that is, the smaller the focal length, the higher the resolution; 4. The resolution is inversely proportional to the first-order magnification rate; that is, the greater the first-order magnification rate, the higher the resolution.

**Figure 8:**
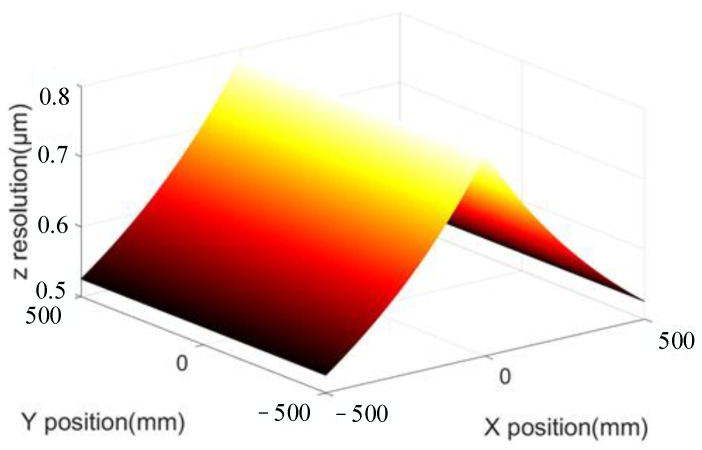
*Z*-resolution of confocal binocular vision system.

**Figure 9:**
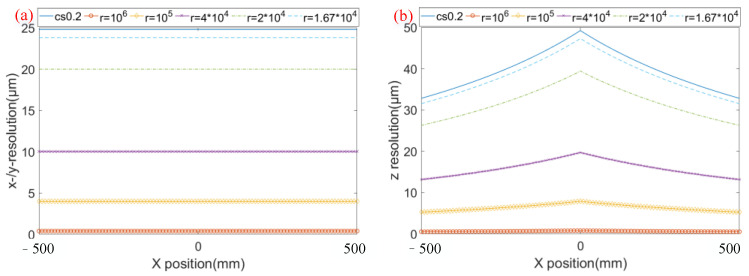
3D resolution of the system with different scan sampling ratios. (**a**) *x*-/*y*-resolution of the system with different scan sampling ratios, (**b**) *z*-resolution of the system with different scan sampling ratios.

Assuming that the focal length of the telephoto lens is 16 mm, the resolution of the matching algorithm has a sampling interval of 0.1, the field of view of the visual system is 1.5 m, the baseline distance is 2 m, the test distance *d* is 2 m, the scanning angle of the galvanometer is 25°, the first-order magnification is 5, the focal length of the scanning lens is 367 mm, the sampling frequency of the data acquisition card is 20 MHz, and the scanning frequency is 20 Hz. The ratio between the sampling frequency of the data acquisition card and the scanning galvanometer is called the scanning sampling ratio, that is, *r* = *n*/*m*. Through calculation, the resolution of the system in the *x*-direction is 0.4 μm, and the resolution in the *y*-direction is also 0.4 μm. The *z*-direction resolution of the confocal binocular vision system is shown in Figure 8. It can be seen that, compared with the traditional vision system, the resolution of the vision measurement system based on confocal scanning is increased by more than 50 times under the same telephoto lens. When the optical system remains unchanged, the resolution of the system increases with the increase in the scan sampling ratio. Since the *x*- and *y*-direction resolutions of the system are consistent in the full field of view, and the *z*-direction resolution is consistent at the same *x* position, the relationship between the system resolution and the scan sampling ratio is shown at *y* = 0, as shown in Figure 9. Among them, cs0.2 represents the resolution of the traditional vision system with a sampling interval of 0.2 μm. As can be seen from the figure, as long as the scanning sampling ratio is greater than 1.6 × 10^4^, the resolution of the vision measurement system based on confocal scanning is theoretically superior to that of the traditional vision system. When the scanning sampling ratio is greater than 10^5^, the resolution of the system can be broken down to less than 10 μm, and with the increase in the scanning sampling ratio, the theoretical resolution of the system will increase proportionally.

**Figure 10:**
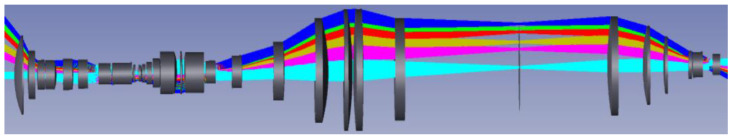
Confocal scanning imaging vision system based on telecentric connection.

However, as can be seen from the schematic diagram, the function of the photographic lens is to ensure the FOV and measurement distance. In order to ensure that the public FOV is not less than 1.5 m × 1.5 m, the diameter of the FOV of the photographic lens is designed to be not less than 3.5 m, which also leads to the relatively large diameter of the image plane of the photographic lens. Therefore, if we use traditional optical design methods to design the entire optical part to achieve the fusion of vision technology and confocal technology, the aperture of some optical lenses in the instrument will be too large. To solve this problem, we designed the lens in the instrument as a telecentric lens, as shown in Figure 10. This can effectively reduce the aperture of the lens, and the aperture of the lens can be limited to less than 180 mm by a certain design method. In the design, it is necessary to ensure that the FOV and pupil match between the lenses so as to make full use of the performance of each lens.

## 3. Test Methods and Experiments

### 3.1. Experimental Equipment

**Figure 11:**
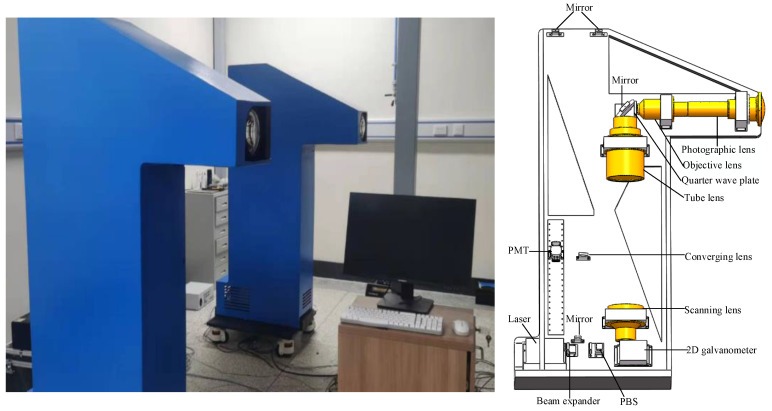
Equipment diagram of a vision system based on confocal scanning imaging.

Figure 11 shows the binocular and monocular vision equipment used for confocal scanning imaging. The two monocular vision systems were placed in parallel. The baseline distance was 2 m, the test distance was 2.5 m, and the public FOV exceeded 1.5 m × 1.5 m. The optical lenses used in the equipment were designed independently. The F-number of the photographic lens was 2, and the focal length was 16 mm. According to the principles of pupil matching and FOV matching, the numerical aperture of the objective lens was set to 0.25, and the focal length was designed to be 50 mm. To increase the freedom of the optical path placement, the focal length of the tube lens was designed to be 375 mm, and the diameter of the incident pupil was designed to be 26 mm to match the pupil. To match the FOV and reduce design difficulty, the focal length of the scanning lens was designed to be 225 mm, and the pupil diameter was 15.6 mm. Owing to the 2D galvanometer, the convergent lens was a converging beam on the axis without an off-axis incident beam; therefore, its diameter was designed to be 20 mm.

### 3.2. Resolution Measuring Equipment and Methods

**Figure 12:**
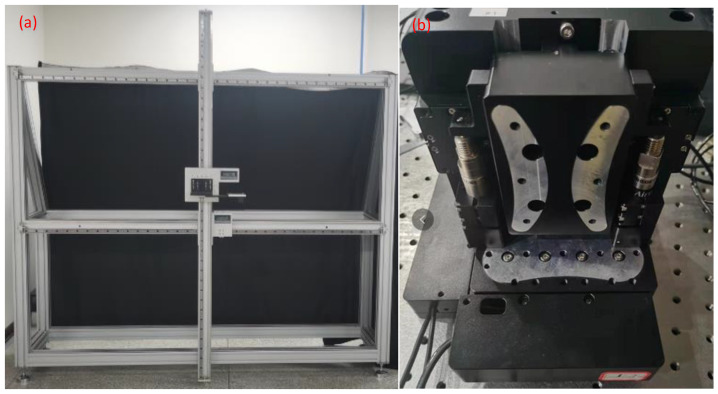
Test system for field of view and resolution. (**a**) Aluminum frame with high-precision and large-stroke 2-D guide rail, and (**b**) 3D high-precision nano-displacement platform.

To verify the resolution of the proposed method in an FOV of no less than 1.5 m × 1.5 m, the size of the object to be measured must be at least 1.5 m × 1.5 m, and a micron-scale 3D displacement must be able to be generated. Controlling such small displacements precisely on a large object is challenging. Hence, a high-precision large-stroke 2D guide rail frame was used to replace large objects, and a high-precision nano-displacement platform was used to achieve high-precision and controllable 3D displacement, as shown in Figure 12. The large 2D guide rail frame was composed of a guide rail and an aluminum alloy frame. The length of each guide rail was 2 m, and a small platform that could shift freely was placed on the guide rail; a magnetic scale was added to the small platform and guide rail to read the position of the small platform. The high-precision micro-displacement platform was a nanometer-scale 3D displacement platform that could exhibit 3D motions in intervals of 50 nm and propagate up to 100 µm.

The experiment was carried out in the laboratory environment and the main measurement conditions were as follows: 1. During the experiment, it is necessary to ensure that the vibration is very small, so a certain vibration isolation condition is required, and the measurement error caused by the fluctuation of the instrument was not more than 1 μm. 2. The test object must have high reflectivity to ensure that the image of the tested object has a high signal-to-noise ratio, so the surface of the object was sprayed with micro-bead reflective film.

After the developed instrument was fixed, the 2D guide rail frame was placed 2.5 m away from the instrument; the center of the frame was located at the center of the instrument’s FOV, and the FOV and resolution were subsequently tested. The test methods and steps were as follows:

1. The target with the characteristic structure was placed on the displacement platform, and the displacement platform was placed in the center of the 2D guide rail frame, which is the center of the FOV of the developed instrument; the reading of the grating ruler was recorded at this time.
2. The position of the small target was adjusted such that the target could be clearly imaged by the developed instrument, and the image of the target was recorded at this time (A).
3. A high-precision micro-displacement platform was used to drive the small target to generate a certain amount of micro-displacement (a μm) along the *x*-direction, and an image (B) of the target was recorded at this time.
4. The pixel movement of images B and A was calculated using a Zero-normalized cross-correlation (ZNCC)-based image-matching algorithm.
5. Steps 3 and 4 were repeated. If the signs of pixel displacement measured repeatedly are the same, then this implies that the resolution of the measuring instrument in the *x*-direction is no less than a μm.
6. The *y*- and *z*-direction resolutions of the instrument at the center of the FOV were tested using the same method.
7. The position of the target was changed, and the distance between the target and the center of the FOV was ensured to exceed 0.75 m. Steps 2 to 6 were repeated.

By performing the steps above, the resolution of the system could be determined within a 1.5 m × 1.5 m FOV.

### 3.3. Experiments

#### 3.3.1. Resolution and Field of View Test Experiment

First, the resolution at the center of the FOV was tested, and the target was placed at the center of the FOV. At this time, the magnetic scale read (953.2 mm, 1072.1 mm). The parameters of the left and right visual systems at the center of the FOV were set as follows: the scanning range was approximately 5 mm × 5 mm, and the number of sampling points was 500 × 500. The target was shifted to the right edge of the FOV, and the reading of the magnetic scale was recorded at this time. The distance from the center of the FOV can be calculated as 1707.5 − 953.2 = 754.3 mm. The scanning range was approximately 5 mm × 5 mm, and the number of sampling points was 500 × 500. Similarly, the target was shifted to the upper edge of the FOV, the reading of the magnetic scale was recorded, and the moving distance can be calculated as 1832.5 − 1072.1 = 760.4 mm. The scanning range was approximately 5 mm × 5 mm, and the number of sampling points was 500 × 500. The resolution was tested at only three locations, owing to the circular symmetry of the monocular vision system. Therefore, the actual FOV was 1.521 m × 1.509 m. Finally, the target shifted to the bottom left corner of the FOV, and the distance from the center of the FOV was exactly 760.4 mm and 754.3 mm.

**Figure 13:**
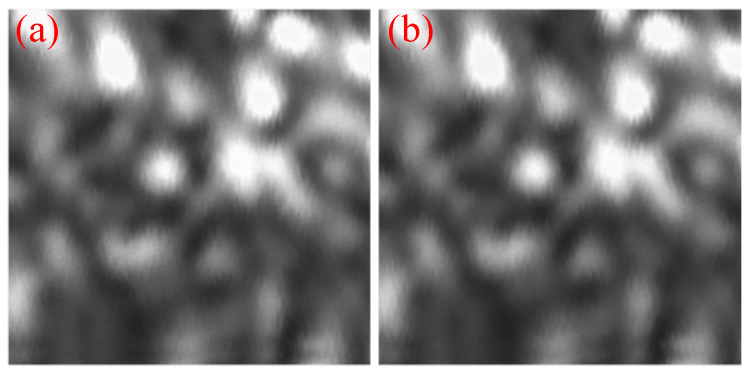
Image of the target. (**a**) Image before moving; (**b**) image after moving 2.5 μm.

At the center and edge of the FOV, a high-precision displacement platform was used to realize displacements at intervals of 2.5 μm in the *x*- and *y*-directions and at intervals of 6 μm in the *z*-direction. The movement was repeated six times for each position and direction, and the calculation results are listed in Table 2, Table 3, Table 4, Table 5, Table 6 and Table 7. Figure 13 shows an image of the target obtained using the left visual system. Figure 13a,b show the images before and after the object shifted, respectively. Almost no visual change was indicated from the figures; therefore, the imaging results are not shown in subsequent tests. Table 2, Table 3, Table 4 and Table 5 show that for the left and right visual systems, when the target shifted monotonically along the *x*- and *y*-directions at intervals of 2.5 μm, the symbols of pixel movement amount obtained by the matching algorithm based on ZNCC were the same, both in the center and at the edge of the FOV. That is, when the target propagated 2.5 μm along the *x*- or *y*-direction, the vision system was able to recognize this displacement. This indicates that the resolution of the developed system can reach 2.5 μm in the *x*- and *y*-directions. Although the resolution was measured at only three locations, one can conclude that the resolution of the system in the *x*- or *y*-direction is 2.5 μm in the full FOV because the system is circularly symmetric. As shown in Table 6 and Table 7, the resolution of the left visual system in the three FOVs in the *z*-direction can reach 6 μm, but the right visual system cannot distinguish this displacement at the right edge of the FOV. This is because the target is near the optical axis of the right visual system at this time; therefore, it cannot be recognized, which is consistent with the simulation results presented in Section 2.1. Although the right visual system cannot recognize this displacement, the left visual system can. Therefore, based on the definition of the resolution of the visual system, the *z*-direction resolution of the visual system can reach 6 μm. Furthermore, because the system is symmetrical, the *z*-resolution of the system in the full FOV can reach 6 μm. Therefore, the resolution of the visual system in the full FOV (1.521 m × 1.509 m) can reach 2.5, 2.5, and 6 μm in the *x*-, *y*-, and *z*-directions, respectively. When the measurement field of view is 1.52 m × 1.51 m, the measurement time is 43.7 s.

According to Table 2 and Table 4, the average pixel displacement of the left system in the *x*- and *y*-directions can be calculated, and the results are 0.1841 and 0.1667, respectively. The actual displacement corresponding to them is 2.50 μm. Through simple calculation, the actual displacement per pixel can be obtained as 2.5/0.1841 ≈ 13.5796 μm/pixel and 2.5/0.1667 ≈ 14.9970 μm/pixel. The measurement results of resolution can be calculated, as shown in Table 2 and Table 4. Similarly, the average pixel displacement of the right system in the *x*- and *y*-directions is 0.1829 and 0.1867, so the corresponding displacement per pixel is 13.6687 μm/ pixel and 13.3905 μm/ pixel. The measurement results of resolution can also be calculated, as shown in Table 3 and Table 5. The average pixel displacement of the left system and the right system in the *z*-direction is 0.1468 and 0.1720, and the corresponding displacement is 6 μm, so the corresponding displacement per pixel is 40.8719 μm/pixel and 34.8837 μm/pixel. The measurement results of resolution can also be calculated, as shown in Table 6 and Table 7. We used the mean of the measurement results of the left and right systems as the accurate measurement results, as shown in Table 8, Table 9 and Table 10. These three tables represent precise measurements of displacement in the *x*-, *y*-, and *z*-directions, respectively. Through calculation, the maximum measurement errors of the proposed algorithm in the *x*-, *y*-, and *z*-directions are 1.3946 μm, 1.3210 μm and 3.9541 μm, respectively, and the standard deviations are 0.7259 μm, 0.6677 μm, and 2.2936 μm.

#### 3.3.2. Comparative Experiments

To further illustrate the performance of our technique, we perform comparative experiments in this section. The type of vision-measuring instrument owned by our laboratory is FARO cobalt. The measuring field of view of the instrument is 260 mm × 200 mm, and the testing distance is 505 mm, which cannot reach the corresponding measuring field of view and distance of our method. In fact, the resolution is directly related to the test distance and field of view; the closer the test distance and the smaller the field of view, the higher the resolution of the visual system. Therefore, we do not directly use resolution for comparison but use relative resolution (resolution/field of view), just like the resolution indicators given by some commercial vision instruments.

**Figure 14:**
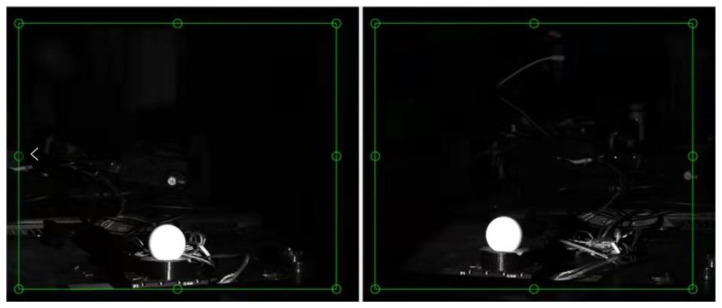
Image of the round ball.

Due to the different illumination methods, we could not use the previous target as the test object for the FARO instrument. So, in this experiment, we used the standard round ball as the test object, and the resolution test procedure was the same as that in Section 3.2. In order to increase the reliability of the experiment, the software of the FARO instrument was used to calculate the spherical center, and the resolution of the system was judged by the changing law of the position of the spherical center. In this experiment, we only tested the resolution at the center and edge of the field of view. In addition, according to the previous analysis, the resolution of the visual system in the *x*-direction and *y*-direction is the same or similar, so only the resolution of the system in the *x*- and *z*-direction is tested in this experiment. The round ball captured by the system is shown in Figure 14. The FARO instrument takes about 13 s to complete a measurement. A high-precision displacement platform was used to realize displacements at intervals of 2.5 μm in the *x*-direction and at intervals of 5 μm in the *z*-direction. The movement was repeated six times for each position and direction, and the calculation results are listed in Table 11 and Table 12. Table 11 shows that for the FARO visual system, when the target shifted monotonically along the *x*-direction at intervals of 2.5 μm, the vision system was able to recognize this displacement. That is, its relative resolution is 2.5/260,000 ≈ 0.0000096 × FOV. Its relative resolution in the *y*-direction is also approximately equal to this value. This indicates that the relative resolution of the FARO system can reach 0.0000096 × FOV in the *x*- and *y*-directions. Similarly, it can be seen from Table 12 that for the FARO vision system, its relative resolution in the *z*-direction is 5/260,000 ≈ 0.000019 × FOV. For our system, the relative resolution in the *x*- and *y*-directions is 2.5/1,500,000 ≈ 0.0000017 × FOV and the relative resolution in the *z*-direction is 6/1,500,000 = 0.000004 × FOV. In other words, our system has 5.8 times better resolution in the *x*- and *y*-directions and 4.75 times better resolution in the *z*-direction compared to the FARO system.

#### 3.3.3. Deformation Test Experiment

**Figure 15:**
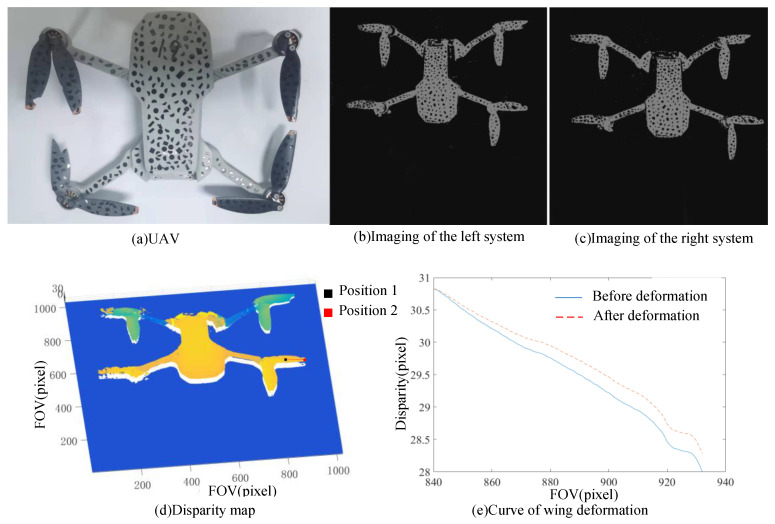
UAV test results. (**a**) The UAV, (**b**) imaging results of the left system, (**c**) imaging results of the right system, (**d**) disparity map, and (**e**) curve of wing deformation.

To further demonstrate the practicability of the method studied in this paper, UAV was taken as the measurement object. One end of the wing of the drone was fixed, and the other end was fixed on the displacement platform. When the displacement table moved axially, the wing appeared to have continuous deformation due to the elasticity of the wing. The UAV was placed in the center of the field of view, and the confocal vision system was used to image the UAV. The results are shown in Figure 15b,c. Then, using the semi-global stereo matching algorithm, the parallax map of the UAV can be calculated, as shown in Figure 15d. It can be seen from the parallax map that there are some mismatching points, which are mainly caused by occlusion, but this is not the content of this paper.

When the displacement table moved 6 μm axially, one end of the wing moved 6 μm. Since the other end of the wing was fixed, the wing underwent continuous deformation. When measuring continuous deformation, the deformation can be measured point by point, or it can be obtained by measuring several points and fitting. Here, in order to save time, we chose two positions for calculation, which are defined as positions 1 and 2, as shown in Figure 15d. The size of each position is 100 px × 100 px. Since position 1 is far away from the displacement table and position 2 is on the displacement table, the deformation at position 2 should be greater than that at position 1. It should be noted that measuring only once may not be accurate enough due to the effects of vibration and noise, so we measured five times after deformation. We took out part of the images corresponding to position 1 and position 2 and then used the image-matching algorithm to match them with the measured images before deformation, respectively, and calculated the pixel displacement, as shown in Table 13. As can be seen from Table 13, the five calculation results at position 1 are different signs, indicating that the deformation at this position cannot be distinguished, so the deformation there is set to 0. The calculation results at position 2 are of the same sign, so it is believed that the deformation there can be distinguished. As can be seen from Table 13, the average pixel movement is 0.278. Considering that the wing deformation is continuous, it is assumed that the deformation conforms to a quadric surface, so the wing deformation diagram can be obtained through fitting, as shown in Figure 15e. To be more intuitive, only one line from position 1 to position 2 is shown. Therefore, the developed system can effectively identify the small continuous deformation in the axial direction of 6 μm, which is consistent with the resolution experimental results.

## 4. Conclusions

In this paper, we propose a new large-field and high-resolution measurement technology—a vision measurement technology based on confocal scanning imaging. Firstly, we built a 3D resolution model of the visual system, through which we analyzed the factors that affect the resolution of the visual system, especially the 3D resolution of the parallel optical axis binocular vision system. Then, the resolution model was used to find a way to improve the resolution of the visual system. Combining confocal scanning imaging technology with vision technology, confocal scanning imaging can effectively compress the sampling interval and achieve a breakthrough in resolution. Finally, the corresponding optical system was designed and the field of view and resolution were measured. The test results show that when the test distance is 2.5 m, the field of view of the developed system can reach 1.521 m × 1.509 m, and the three-dimensional resolutions are 2.5 μm, 2.5 μm, and 6 μm, respectively.

In theory, the smaller the sampling interval, the higher the resolution; however, due to noise, vibration, and other reasons, the resolution cannot be infinitely improved. Therefore, it is necessary to analyze the effects of noise and vibration in order to further improve the resolution. And our technology requires scanning of objects, so the measurement efficiency is lower than traditional vision technology. Therefore, it is necessary to research how to improve the measurement efficiency.

## Figures and Tables

**Table 1:** Resolution of the vision system.

Number of Moves		1	2	3	4	5
*x*-resolution	Displacement (pixel)	0.5172	0.5044	0.5026	0.5045	0.5038
*y*-resolution	Displacement (pixel)	0.5424	0.5369	0.5386	0.5370	0.5450
*z*-resolution	Displacement (pixel)	0.5499	0.5682	0.5631	0.5659	0.5641

**Table 2:** *X*-direction resolution of the left system at the center and edge of the FOV.

Number of Moves		1	2	3	4	5
Center of FOV	Displacement (pixel)	0.2038	0.0855	0.1771	0.2511	0.1901
	Displacement (μm)	2.7675	1.1611	2.4049	3.4098	2.5815
Upper edge of FOV	Displacement (pixel)	0.1350	0.1032	0.2745	0.1539	0.1965
	Displacement (μm)	1.8332	1.4014	3.7276	2.0899	2.6684
Right edge of FOV	Displacement (pixel)	0.0969	0.2864	0.1835	0.1380	0.2269
	Displacement (μm)	1.3159	3.8892	2.4919	1.8740	3.0812
Lower left of FOV	Displacement (pixel)	0.1521	0.2113	0.1663	0.2632	0.1857
	Displacement (μm)	2.0655	2.8694	2.2583	3.5742	2.5217

**Table 3:** *X*-direction resolution of the right system at the center and edge of the FOV.

Number of Moves		1	2	3	4	5
Center of FOV	Displacement (pixel)	−0.1856	−0.0768	−0.2059	−0.2356	−0.2727
	Displacement (μm)	2.5369	1.0498	2.8144	3.2204	3.7275
Upper edge of FOV	Displacement (pixel)	−0.2745	−0.1353	−0.2126	−0.0850	−0.1773
	Displacement (μm)	3.7521	1.8494	2.9060	1.1618	2.4235
Right edge of FOV	Displacement (pixel)	−0.1369	−0.2539	−0.1566	−0.1234	−0.1482
	Displacement (μm)	1.8713	3.4705	2.1405	1.6867	2.0257
Lower left of FOV	Displacement (pixel)	−0.1022	−0.1689	−0.2698	−0.2553	−0.1812
	Displacement (μm)	1.3969	2.3086	3.6878	3.4896	2.4768

**Table 4:** *Y*-direction resolution of the left system at the center and edge of the FOV.

Number of Moves		1	2	3	4	5
Center of FOV	Displacement (pixel)	−0.1473	−0.0567	−0.2336	−0.1577	−0.1250
	Displacement (μm)	2.2091	0.8503	3.5033	2.3650	1.8746
Upper edge of FOV	Displacement (pixel)	−0.2036	−0.1583	−0.0921	−0.2123	−0.1752
	Displacement (μm)	3.0534	2.3740	1.3812	3.1839	2.6275
Right edge of FOV	Displacement (pixel)	−0.1033	−0.1851	−0.1425	−0.2036	−0.2567
	Displacement (μm)	1.5492	2.7759	2.1371	3.0534	3.8497
Lower left of FOV	Displacement (pixel)	−0.2231	−0.1187	−0.1361	−0.2482	−0.1555
	Displacement (μm)	3.3458	1.7801	2.0411	3.7223	2.3320

**Table 5:** *Y*-direction resolution of the right system at the center and edge of the FOV.

Number of Moves		1	2	3	4	5
Center of FOV	Displacement (pixel)	−0.2020	−0.1126	−0.2859	−0.1743	−0.2216
	Displacement (μm)	2.7045	1.5078	3.8283	2.3340	2.9673
Upper edge of FOV	Displacement (pixel)	−0.3025	−0.2718	−0.1478	−0.2356	−0.0636
	Displacement (μm)	4.0506	3.6395	1.9791	3.1548	0.8516
Right edge of FOV	Displacement (pixel)	−0.2985	−0.1356	−0.1026	−0.1461	−0.1510
	Displacement (μm)	3.9971	1.8158	1.3739	1.9564	2.0220
Lower left of FOV	Displacement (pixel)	−0.1993	−0.2512	−0.1386	−0.1915	−0.1011
	Displacement (μm)	2.6687	3.3637	1.8559	2.5643	1.3538

**Table 6:** *Z*-direction resolution of the left system at the center and edge of the FOV.

Number of Moves		1	2	3	4	5
Center of FOV	Displacement (pixel)	−0.0431	−0.1157	−0.2651	−0.0968	−0.1711
	Displacement (μm)	1.7616	4.7289	10.8351	3.9564	6.9932
Upper edge of FOV	Displacement (pixel)	−0.1164	−0.1562	−0.2126	−0.0215	−0.2243
	Displacement (μm)	4.7575	6.3842	8.6894	0.8787	9.1676
Right edge of FOV	Displacement (pixel)	−0.1816	−0.1531	−0.1082	−0.2347	−0.1013
	Displacement (μm)	7.4223	6.2575	4.4223	9.5926	4.1403
Lower left of FOV	Displacement (pixel)	−0.1210	−0.0852	0.1332	0.0878	−0.1026
	Displacement (μm)	\	\	\	\	\

**Table 7:** *Z*-direction resolution of the right system at the center and edge of the FOV.

Number of Moves		1	2	3	4	5
Center of FOV	Displacement (pixel)	0.0668	0.1425	0.2041	0.0811	0.2988
	Displacement (μm)	2.3302	4.9709	7.1198	2.8291	10.4233
Upper edge of FOV	Displacement (pixel)	0.1778	0.2157	0.2691	0.1151	0.1373
	Displacement (μm)	6.2023	7.5244	9.3872	4.0151	4.7895
Right edge of FOV	Displacement (pixel)	0.1132	−0.1054	−0.0589	0.1533	0.0637
	Displacement (μm)	\	\	\	\	\
Lower left of FOV	Displacement (pixel)	0.1882	0.2125	0.1059	0.1282	0.2371
	Displacement (μm)	6.5651	7.4128	3.6942	4.4721	8.2709

**Table 8:** Accuracy measurements of *x*-direction resolution at the center and edge of the FOV.

Number of Moves		1	2	3	4	5
Center of FOV	Displacement (μm)	2.6522	1.1054	2.6097	3.3151	3.1545
Upper edge of FOV	Displacement (μm)	2.7927	1.6254	3.3168	1.6259	2.5459
Right edge of FOV	Displacement (μm)	1.5936	3.6798	2.3162	1.7804	2.5535
Lower left of FOV	Displacement (μm)	1.7312	2.5890	2.9731	3.5319	2.4993

**Table 9:** Accuracy measurements of *y*-direction resolution at the center and edge of the FOV.

Number of Moves		1	2	3	4	5
Center of FOV	Displacement (μm)	2.4570	1.1791	3.6658	2.3495	2.4210
Upper edge of FOV	Displacement (μm)	3.5520	3.0068	1.6801	3.1693	1.7396
Right edge of FOV	Displacement (μm)	2.7731	2.2959	1.7555	2.5049	2.9359
Lower left of FOV	Displacement (μm)	3.0073	2.5719	1.9485	3.1433	1.8429

**Table 10:** Accuracy measurements of *z*-direction resolution at the center and edge of the FOV.

Number of Moves		1	2	3	4	5
Center of FOV	Displacement (μm)	2.0459	4.8499	8.9775	3.3927	8.7082
Upper edge of FOV	Displacement (μm)	5.4799	6.9543	9.0383	2.4469	6.9786
Right edge of FOV	Displacement (μm)	7.4223	6.2575	4.4223	9.5926	4.1403
Lower left of FOV	Displacement (μm)	6.5651	7.4128	3.6942	4.4721	8.2709

**Table 11:** *X*-direction resolution of the FARO system at the center and edge of the FOV.

Number of Moves		1	2	3	4	5
Center of FOV	Displacement (μm)	2.00	4.00	1.00	4.00	3.00
Edge of FOV	Displacement (μm)	4.00	4.00	1.00	5.00	2.00

**Table 12:** *Z*-direction resolution of the FARO system at the center and edge of the FOV.

Number of Moves		1	2	3	4	5
Center of FOV	Displacement (μm)	7.00	4.00	6.00	8.00	5.00
Edge of FOV	Displacement (μm)	3.00	9.00	3.00	2.00	4.00

**Table 13:** The deformation test of UAV wing.

Number of Measurements			1	2	3	4	5
Position 1	Deformation (pixel)	left system	−0.1133	0.1516	0.1308	−0.1176	0.0532
		right system	0.1854	0.0887	−0.1023	−0.2001	0.1682
Position 2	Deformation (pixel)	left system	−0.1512	−0.0885	−0.1362	−0.2431	−0.1147
		right system	0.0989	0.1035	0.0876	0.1553	0.2113

## Data Availability

The data that support the findings of this study are available from the corresponding author, upon reasonable request.
